# Coronary MR angiography using image‐based respiratory motion compensation with inline correction and fixed gating efficiency

**DOI:** 10.1002/mrm.26678

**Published:** 2017-03-20

**Authors:** Markus Henningsson, Jouke Smink, Gerald van Ensbergen, Rene Botnar

**Affiliations:** ^1^ Division of Biomedical Engineering and Imaging Sciences, King's College London London, United Kingdom; ^2^ Philips Healthcare Best The Netherlands

**Keywords:** Motion compensation, coronary magnetic resonance angiography, image‐based navigation

## Abstract

**Purpose:**

The purpose of this study was to evaluate a new inline motion compensation approach called *image‐based navigation with Constant Respiratory efficiency UsIng Single End‐expiratory threshold* (iNAV‐CRUISE) for coronary MR angiography (CMRA).

**Methods:**

The CRUISE gating technique was combined with iNAV motion correction and implemented inline for motion‐compensated CMRA on a 1.5 Tesla scanner. The approach was compared to conventional diaphragmatic navigator gating (dNAVG) in 10 healthy subjects. The CMRA images were compared for vessel sharpness and visual score of the right coronary artery (RCA), left anterior descending artery (LAD), left circumflex, and scan time.

**Results:**

The scan time was similar between the methods (dNAV_G_: 6:32 ± 1:09 vs. iNAV‐CRUISE: 6:58 ± 0:17, *P* = not significant). However, the vessel sharpness of the RCA (dNAV_G_: 60.2 ± 10.1 vs. iNAV‐CRUISE: 71.8 ± 8.9, *P* = 0.001) and LAD (dNAV_G_: 58.0 ± 8.0 vs. iNAV‐CRUISE: 67.4 ± 7.1, *P* = 0.008) were significantly improved using iNAV‐CRUISE. The visual score of the RCA was higher using iNAV‐CRUISE compared to dNAV_G_ (dNAV_G_: 3,4,3 vs. iNAV‐CRUISE: 4,4,3, *P* < 0.01).

**Conclusion:**

The iNAV‐CRUISE approach out‐performs the conventional respiratory motion compensation technique in healthy subjects. Although scan time was comparable, the image quality was improved using iNAV‐CRUISE. Magn Reson Med 79:416–422, 2018. © 2017 The Authors Magnetic Resonance in Medicine published by Wiley Periodicals, Inc. on behalf of International Society for Magnetic Resonance in Medicine. This is an open access article under the terms of the Creative Commons Attribution License, which permits use, distribution and reproduction in any medium, provided the original work is properly cited.

## INTRODUCTION

Whole‐heart coronary MR angiography (CMRA) allows for noninvasive and ionizing radiation free detection of lumen‐narrowing coronary artery disease (CAD). Nevertheless, CMRA in patients with CAD remains limited due to the long scan times and unpredictable and sometimes nondiagnostic CMRA image quality 1. The most common image degradation in CMRA is caused by image blurring and ghosting from respiratory motion. This is due to the necessity of acquiring high‐resolution whole‐heart CMRA during free‐breathing. The conventional strategy for respiratory‐motion compensation involves using a diaphragmatic one‐dimensional navigator (dNAV), measuring the displacement of the lung–liver interface in foot–head (FH) direction, which is the predominant motion direction 2. This approach allows for tracking of the respiratory motion of the heart, typically using an assumed linear scaling factor between the translational FH motion of the diaphragm and the heart 2, 3. However, the respiratory induced motion of the heart has been shown to be nonrigid and 3D 4. This can be addressed by respiratory gating whereby the respiratory navigator information is used to accept CMRA data only if it is in a small motion window, typically centered on end‐expiration. Although respiratory gating effectively reduces the motion to a narrower range, which can be approximated by linear translation, it has the adverse effect of prolonging the scan time considerably, often by a factor of two or more. Such accept–reject gating strategies with fixed navigator windows also have the drawback of unpredictable scan times because gating efficiency may vary considerably between patients. In the case of patients with irregular breathing or respiratory drift, the respiratory gating efficiency even can change during a scan, which adds further uncertainty to the total scan duration. Alternative strategies have been proposed, which reduce the scan time overhead and the uncertainty introduced by respiratory gating, such as diminishing variance algorithm 5 or phase ordering with automatic window selection (PAWS) 6.

In the last decade, a number of navigator techniques have been described that allow direct measurement and correction of respiratory‐induced motion of the heart. These include self‐navigation (selfNAV), which extracts the motion information from the CMRA data itself 7, 8, 9; and image‐based navigation (iNAV), for which 2D 10, 11 or 3D 12 real‐time images are used to estimate bulk respiratory motion of the heart. In addition to directly tracking respiratory motion of the heart, selfNAV simplifies CMRA ease of use compared to other navigator approaches because no dedicated navigator scan planning is necessary 8, 13. Compared to 1D selfNAV, which is a projection of the entire field of view (FOV), including static chest wall, iNAV allows spatial separation of static and moving structures. Recently, a 2D iNAV has been proposed, which combines the advantages of iNAV navigation (separation of moving and static structures) with selfNAV (ease of use) in which the navigator images are generated by spatially encoding the start‐up echoes of a balanced steady‐state free precession (bSSFP) sequence 14. With this approach, translational motion of the heart can be directly measured and corrected in FH and left–right (LR) direction. To compensate for nonrigid respiratory motion, which may occur between end‐inspiration and end‐expiration, respiratory gating was implemented using an external respiratory bellows signal aiming to limit motion to approximately translational motion around end‐expiration. However, improved performance, ease of use, and predictable scan time are likely to be achieved using the iNAV to respiratory gate the CMRA scan rather than an external surrogate signal.

The purpose of this study was to evaluate a new inline motion compensation approach called *iNAV with Constant Respiratory efficiency UsIng Single End‐expiratory threshold (iNAV‐CRUISE) for coronary MR angiography*. The proposed approach was compared to the conventional diaphragmatic navigator motion correction technique in healthy volunteers.

## METHODS

The studies were approved by the local ethics committee, and all participants provided written informed consent. All experiments were performed on a 1.5 Tesla clinical scanner (Philips Healthcare, Best, The Netherlands) using a 32‐channel cardiac coil.

### Image Navigator Acquisition and Motion Correction

The acquisition and postprocessing for the iNAV acquisition and motion correction has been previously described 13, 14. In brief, 2D real‐time navigator images are generated by adding phase‐encoding gradients to the 10 startup echoes of a bSSFP sequence, resulting in a 2D projection of the 3D volume in the slice‐encoding direction. Using a coronal CMRA field‐of‐view orientation, with readout in FH direction and phase encoding in LR direction, the iNAV allows 2D motion estimation and correction in these directions. An iNAV region of interest (ROI) centered on the heart can be defined by using the local shim volume, which encompassed the heart. The first acquired iNAV ROI is used as reference, to which all subsequent iNAVs are registered using normalized cross‐correlation, which in turn provides translational motion information. The 2D translational correction was applied to the CMRA k‐space raw data by modulating its phase.

### CRUISE Gating

**Figure 1:**
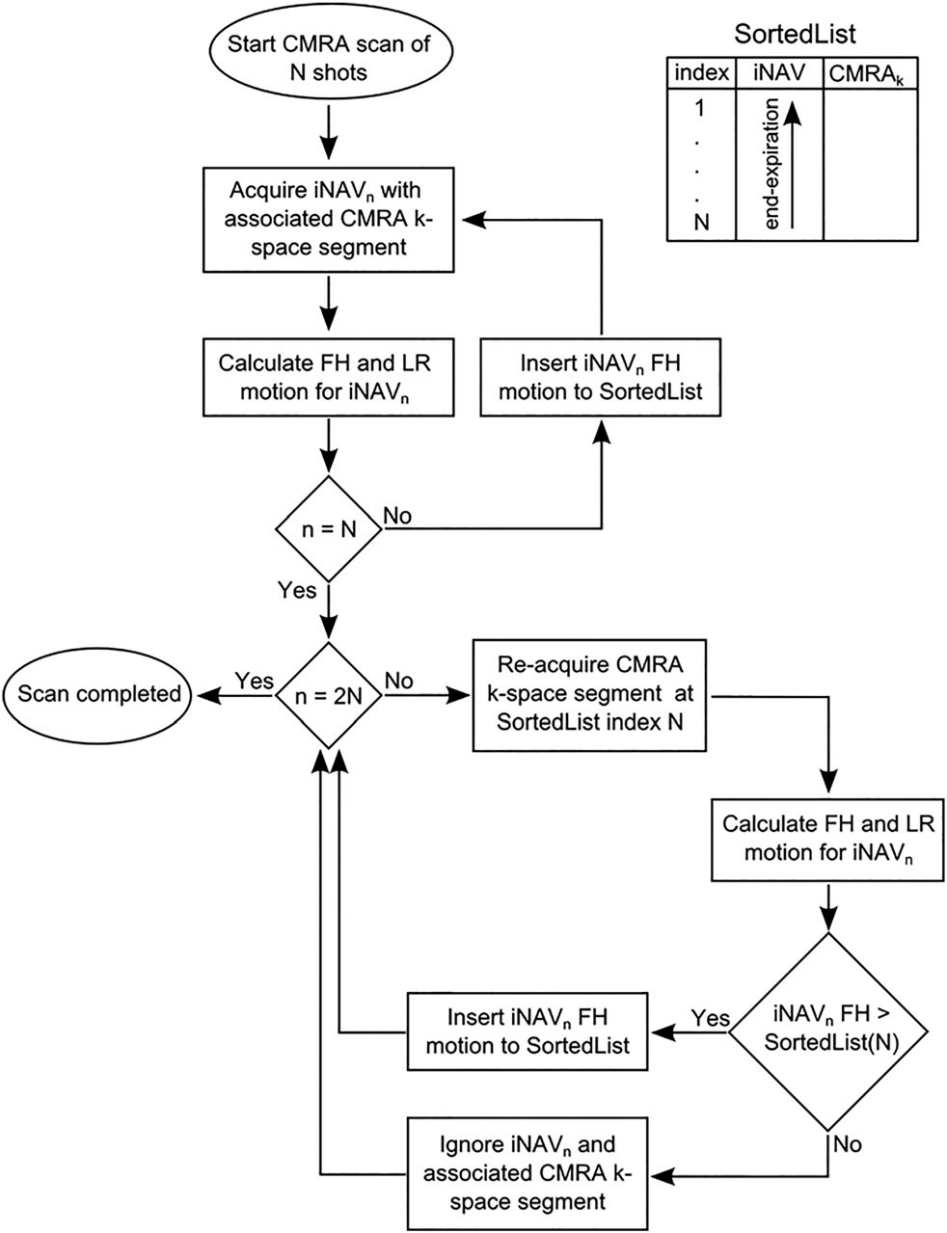
Flowchart of the proposed CRUISE gating algorithm. During the first half of the scan, CMRA k‐space is completely filled, and the corresponding iNAV FH values are used to populate a sorted list. Motion values in the sorted list are ordered such that ascending FH values are associated with a more end‐expiratory position. In the second half of the scan, the k‐space segment associated with the most inspiratory iNAV is re‐measured, and the new iNAV position is added to the sorted list if it is above the temporary gating threshold, which is defined by the most inspiratory iNAV position in the sorted list. CMRA, coronary MR angiography; CRUISE, Constant Respiratory efficiency UsIng Single End‐expiratory threshold; FH, foot–head; iNAV, image‐based navigator; LR, left–right.

**Figure 2:**
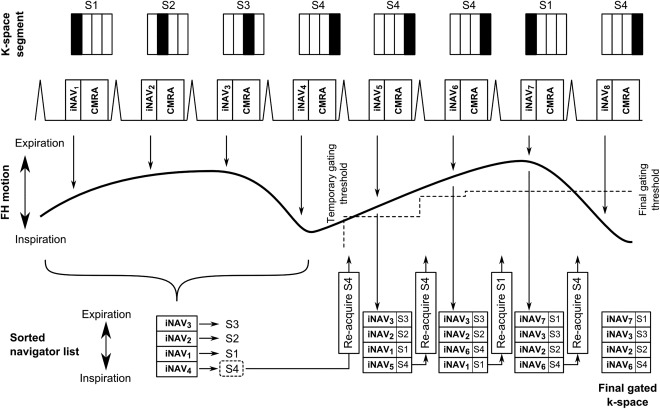
Proposed CRUISE respiratory gating algorithm in a simplified CMRA acquisition, consisting of four k‐space segments (S1–4). The CRUISE approach results in a gradually diminishing motion range as more expiratory positions are obtained and added to the sorted list. If the re‐measured iNAV position is below the gating threshold, as in the last cardiac cycle, the k‐space segment is ignored. CMRA, coronary MR angiography; CRUISE, Constant Respiratory efficiency UsIng Single End‐expiratory threshold; FH, foot–head; iNAV, image‐based navigator; S, segment.

Previous iNAV implementations have utilized respiratory gating in addition to translational correction, either using a separate navigator acquisition 10, 13 or an external respiratory signal 14. In the separate navigator approach, the gating window was fixed around end‐expiration based on a navigator preparation phase 10, 13, whereas the external bellows signal used a pressure meter on the abdomen to determine the respiratory phase and gate to end‐expiration 14. The proposed algorithm enables inline respiratory gating using the motion estimated directly from the iNAVs. The CRUISE‐gating algorithm aims to ensure that the CMRA k‐space segments are acquired in the most end‐expiratory positions. A flowchart of the algorithm is shown in Figure 1. CRUISE assumes a fixed gating efficiency of 50%, meaning that half of the acquired data is discarded and re‐measured. The measured respiratory positions in FH direction are added to a sorted list, in which high values correspond to end‐expiration and low values correspond to end‐inspiration. During the first half of the scan, all CMRA data are stored and the sorted list is populated. During the second half of the scan, the CMRA k‐space segment associated with the lowest value (end‐inspiration) is discarded and re‐measured. This result in a gradually narrowing respiratory motion range because an increasing number of CMRA k‐space segments are acquired closer to end‐expiration. In the second phase of the scan, if a measured FH position is outside the temporary gating window, it is discarded and re‐measured during the following cardiac cycle. A simplified example CMRA acquisition using the proposed CRUISE gating algorithm is illustrated in Figure 2.

### Healthy Volunteer Studies

iNAV‐CRUISE, as outlined in the previous section, was compared to 1D selfNAV (using only the k_0_‐line of k‐space for navigator acquisition) with CRUISE gating (selfNAV‐CRUISE). Additionally, images were acquired with ungated dNAV using a correction factor of 0.6 and two number of signal averages (2NSA) (dNAV‐2NSA) to ensure identical scan efficiency as the CRUISE gated scans. CMRA data was acquired in five healthy volunteers (mean age 32 ± 3 years old, 3 females) using iNAV‐CRUISE, self‐NAV‐CRUISE, and dNAV‐2NSA in a randomized order.

In a second study, the proposed iNAV‐CRUISE was compared to the conventional reference method used for clinical CMRA‐dNAV, with a 0.6 tracking factor and 5‐mm gating window (dNAV_G_). CMRA scans using the two different motion compensation strategies were performed in a randomized order in 10 healthy subjects (mean age = 29 ± 5 years old, 4 females).

The bSSFP CMRA imaging parameters for all healthy volunteer scans included FOV = 300 × 300 × 110 mm^3^, Δx = 1.3 × 1.3 × 1.3 mm^3^, repetition time/echo time = 3.9/1.95 ms, α = 70 °, and parallel imaging acceleration factor = 2.5 (in‐plane phase encoding direction). Electrocardiogram triggering was used to minimize cardiac motion, with subject‐specific trigger delays and acquisition windows. To improve CMRA contrast, T2 prep (echo time = 35 ms) and fat suppression pre‐pulses were used. The nominal scan time, excluding respiratory gating, was 3 minutes and 20 seconds, assuming a heart rate of 60 beats per minute and an acquisition window of 120 ms.

### Data Analysis

All CMRA images were reformatted to visualize the right coronary artery (RCA), left main and left anterior descending artery (LAD), and left circumflex artery (LCX) using dedicated software 15.

To objectively and subjectively assess CMRA image quality, vessel sharpness measurements and visual score were performed on all datasets. Using dedicated software, vessel sharpness was calculated on the first 4 cm of all coronary arteries as a percentage in which 0% equals no edge and 100% equals a step edge 15.

A visual score was used, based on a previous CMRA patient study 16, to qualitatively assess coronary image quality based on the following scale: 0 = coronary artery not visible, 1 = visible but with marked blurring, 2 = visible with moderate blurring, 3 = visible with mild blurring, and 4 = visible with sharp edges. For the healthy volunteer, CMRA data acquired with iNAV and dNAV_G_, each coronary artery (RCA, LAD, and LCX) was scored by one reviewer with 8 years of experience in CMRA.

### Statistical Analysis

All statistical analysis was performed using MatLab (The MathWorks Inc., Natick, MA, USA) statistics toolbox. For the continuous variables vessel sharpness and scan time, a two‐tailed *t* test was performed to evaluate statistical significance. Continuous variables are presented as mean ± standard deviation (SD). For the categorical variable (visual score), a Wilcoxon signed‐rank test was performed to evaluate statistical significance. Categorical variables are presented as median, 75th percentile, and 25th percentile. A *P* value smaller than 0.05 was considered statistically significant. Bonferroni correction was performed on the *P* values of the vessel sharpness and visual scores of the healthy volunteers to account for multiple comparisons, resulting in a significance threshold of 0.05/3 = 0.017.

## RESULTS

### Comparing iNAV‐CRUISE, selfNAV‐CRUISE, and dNAV‐2NSA

Reformatted CMRA images for all five scanned volunteers comparing iNAV‐CRUISE, selfNAV‐CRUISE, and dNAV‐2NSA are shown in Supporting Figure S1. Improved visualization of the coronary arteries was obtained in all volunteers and coronary arteries using iNAV‐CRUISE compared to self‐NAV‐CRUISE. Superior coronary artery visualization was also obtained using iNAV‐CRUISE compared to dNAV‐2NSA in four out of the five healthy volunteers. In one volunteer (number 5) with frequent ectopic beats and irregular breathing, a comparable but poor image quality was observed with both techniques, and no vessel sharpness of the right coronary artery could be obtained with either technique. The vessel sharpness measurements for iNAV‐CRUISE, self‐NAV‐CRUISE, and dNAV‐2NSA are summarized in Supporting Figure S2.

### Comparing iNAV‐CRUISE and dNAVG

**Figure 3:**
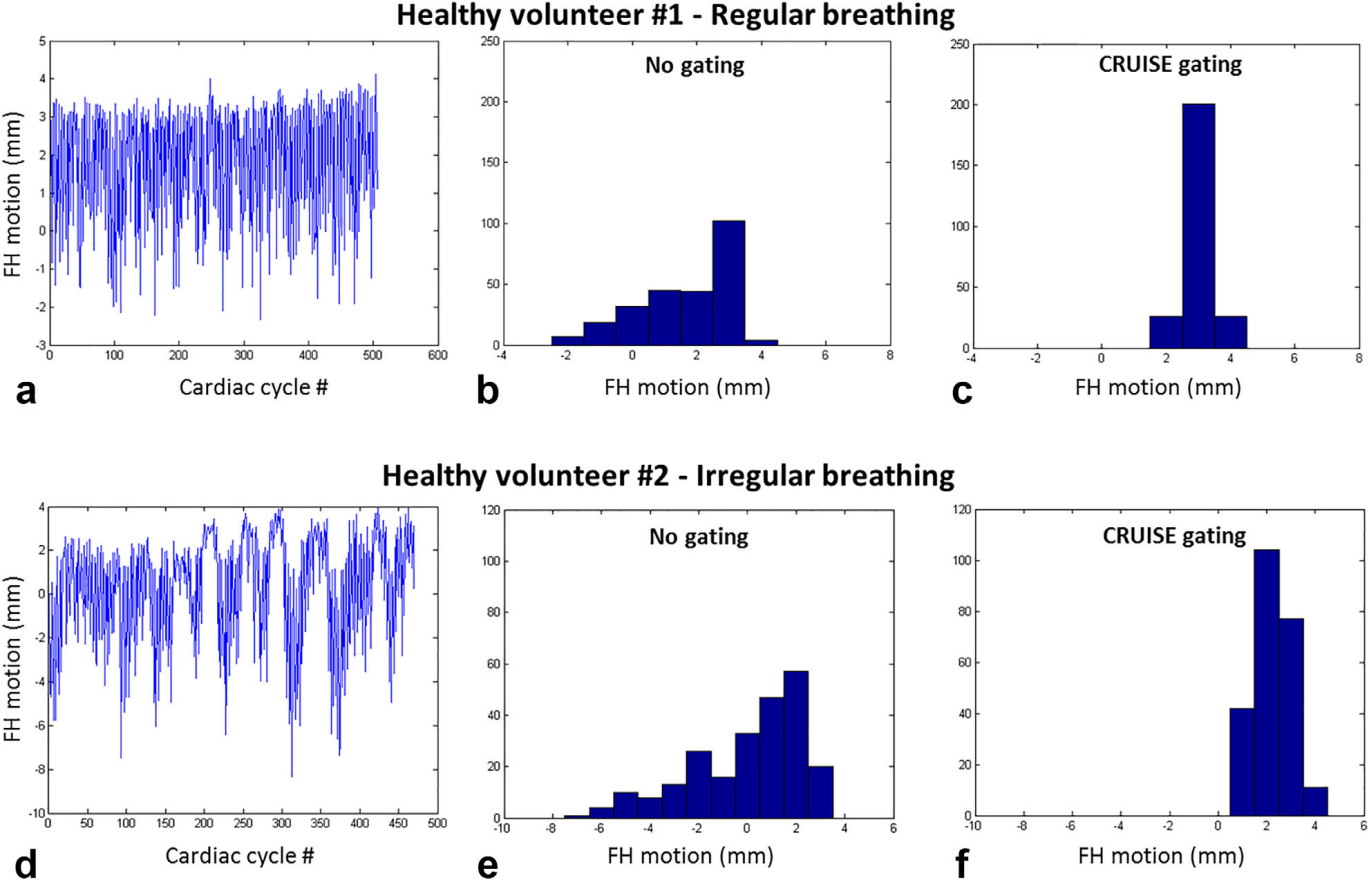
Respiratory motion pattern for two healthy volunteers, one with regular breathing (**a**) and one with irregular breathing pattern (**D**). The histograms of the motion values using no gating (**b, d**) and the proposed iNAV‐CRUISE gating approach (**c, f**), resulting in a narrower motion range in both cases. CRUISE, Constant Respiratory efficiency UsIng Single End‐expiratory threshold; FH, foot–head.

**Figure 4:**
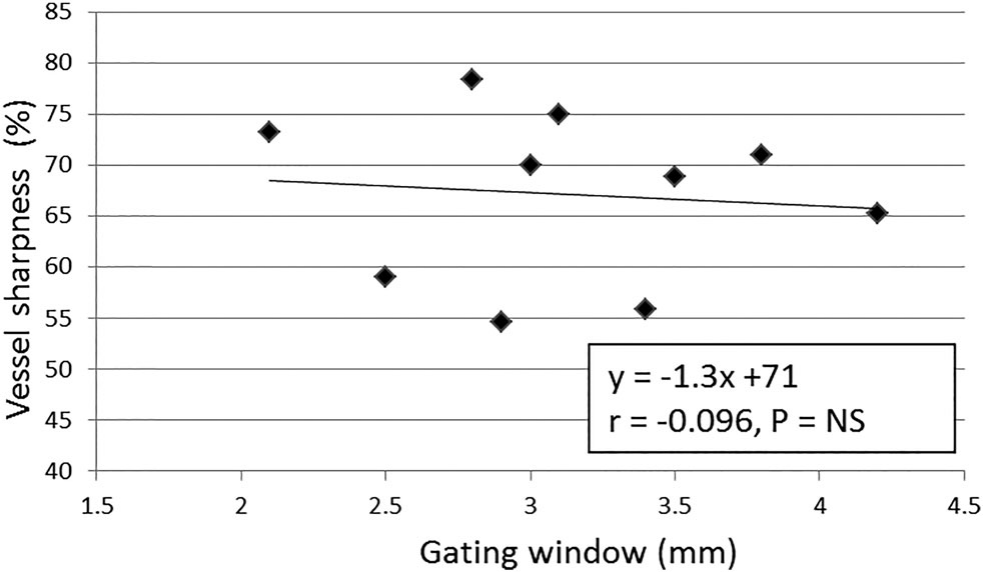
Correlation between the final gating window and vessel sharpness (averaged across the 3 coronary vessels) for 10 healthy subjects.

The mean gating efficiency ± SD using dNAV_G_ was 55.3% ± 10.9%. The final gating window using the proposed CRUISE gating algorithm, averaged over the 10 healthy subjects, was 3.1 ± 0.6 mm. Assuming a diaphragm‐heart respiratory motion correlation of 0.6, this corresponds to a diaphragmatic gating window of just above 5 mm. In comparison, without using the proposed gating algorithm, the motion range of respiratory positions in FH direction was 8.2 ± 2.5 mm across the 10 subjects. This was based on the FH motion values from the first half of the gated iNAV scan. Although the estimated LR motion was not incorporated into the CRUISE gating mechanism, the use of gating reduced the range of LR motion from 1.1 ± 0.5 mm to 0.3 ± 0.3 mm. Respiratory motion patterns for two healthy volunteers, one with regular and one with irregular breathing, are shown in Figures 3 a, d. In the case of irregular breathing, the final gating window is larger, as shown in the distribution of motion values in the gated scan in Figure 3f. A scatterplot of the final gating window and the coronary vessel sharpness, averaged over the three coronary vessels, for all 10 healthy volunteers is shown in Figure 4. Although there was a slight trend toward reduced vessel sharpness with larger gating windows, no statistically significant correlation was found between the variables.

**Figure 5:**
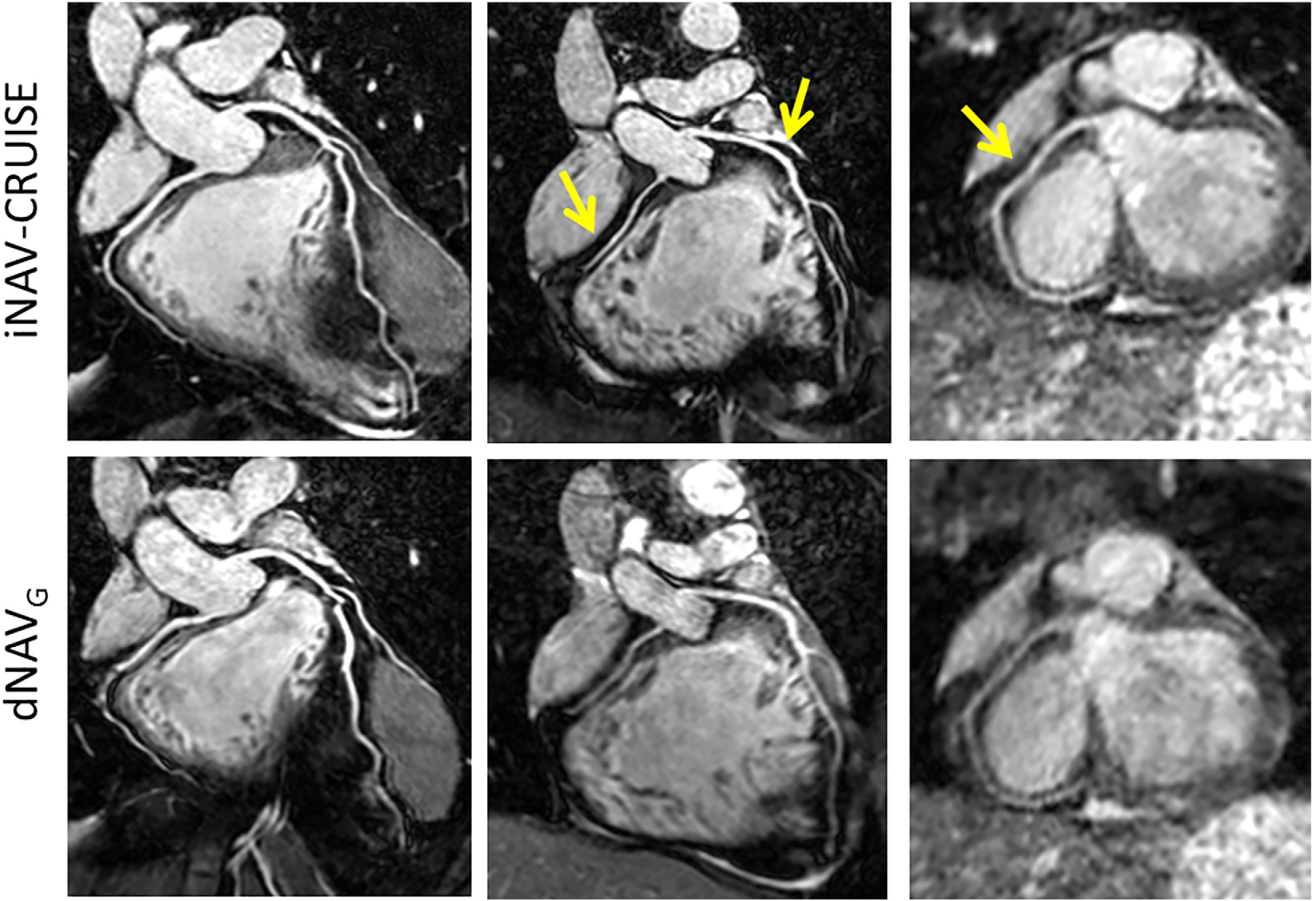
Representative coronary MR angiography reformats acquired in three healthy subjects using iNAV‐CRUISE and dNAV_G_. Arrows highlight coronary arteries with improved sharpness. dNAV_G_, diaphragmatic navigator with gating; iNAV‐CRUISE, image‐navigated Constant Respiratory efficiency UsIng Single End‐expiratory threshold.

Representative CMRA images using iNAV‐CRUISE and dNAV_G_ from three healthy subjects are shown in Figure 5. Although the scan time was similar using dNAV_G_ and iNAV‐CRUISE (dNAV_G_: 6:32 ± 1:09 vs. iNAV‐CRUISE: 6:58 ± 0:17, *P* = Not significant), the vessel sharpness of the RCA (dNAV_G_: 60.2 ± 10.1 vs. iNAV‐CRUISE: 71.8 ± 8.9, *P* = 0.001) and LAD (dNAV_G_: 58.0 ± 8.0 vs. iNAV‐CRUISE: 67.4 ± 7.1, *P* = 0.008) were significantly improved using iNAV‐CRUISE, whereas the difference for LCX (dNAV_G_: 53.5 ± 7.3 vs. iNAV‐CRUISE: 58.1 ± 9.1, *P* = N.S.) did not reach statistical significance. Furthermore, the visual score of the RCA was significantly higher using iNAV‐CRUISE compared to dNAV_G_ (dNAV_G_: 3,4,3 vs. iNAV‐CRUISE: 4,4,3, *P* < 0.01), whereas the LAD (dNAV_G_: 3,4,3 vs. iNAV‐CRUISE: 4,4,3, *P* = N.S.) and LCX (dNAV_G_: 3,3,3 vs. iNAV‐CRUISE: 3,4,3, P = N.S.) were not statistically different.

## DISCUSSION

In this work, we have implemented a new approach for image‐navigated CMRA using inline retrospective respiratory gating with constant gating efficiency and have evaluated it in healthy subjects. We have found that the proposed iNAV‐CRUISE algorithm improves image quality compared to alternative navigator approaches, including 1D selfNAV and diaphragmatic pencil‐beam navigator with identical scan efficiencies. Importantly, this gating approach allows reducing the range of motion values without relying on external sensors 14, which simplify scan setup. Although iNAV‐CRUISE results in a range of respiratory motion, which is variable between subjects, we found no significant correlation between final gating window size and image quality. This suggests that the use of a constant 50% efficiency reduces motion to a respiratory phase, which can be well‐approximated by a translational motion model. Nevertheless, the small number of subjects in this study, most of which had regular breathing, limits generalization of these findings to patients with CAD in whom irregular breathing patterns are more commonly observed.

We found significantly improved image quality for the RCA (visual score and vessel sharpness) and LAD (vessel sharpness) using the proposed iNAV‐CRUISE approach compared to conventional diaphragmatic gating and tracking. Because both approaches on average had similar respiratory gating levels (after accounting for difference in location), this improvement is likely due to the direct and more accurate motion estimation of the iNAV. No significant difference between the two methods was found for the LCX. This may be attributed to the generally lower signal‐to‐noise of the posterior side of the heart, which is further away from the receiver coils. Signal from the adjacent coronary vein also can impede visualization of the LCX. Further studies in patients with congenital heart disease and CAD are underway to investigate it the improved image quality translates into better diagnostic performance.

### Technical Considerations

Compared to previous iNAV implementations using startup echoes of the bSSFP sequence for the iNAV acquisition, the proposed approach does not rely on additional navigator acquisitions 13 or external respiratory bellows 14 for the gating. Instead, the CRUISE gating algorithm was directly based on the calculated iNAV motion values and implemented on the scanner reconstruction computer to enable inline motion compensation. Although recent iNAV approaches have achieved gating efficiencies approaching 100%, with image quality comparable to those obtained with the conventional dNAV method, such techniques typically employ complex, computationally expensive postprocessing algorithms that require manual input 17, 18. The drawbacks of such techniques are a major hurdle for the clinical translation, and actually may reduce ease of use and increase user dependence of CMRA. In contrast, the proposed technique requires no postprocessing or user interaction because all motion compensation can be automated and implemented on the reconstruction computer. Whereas the scan time is doubled using the proposed gating algorithm compared to an ungated scan, it is completely predictable and known before the scan, unlike the conventional dNAV_G_ approach in which the gating window is predefined and the gating efficiency (and subsequently the scan time) is unknown. A major reason that CMRA remains scarcely used in the CAD population is the unpredictable scan time associated with the conventional navigator technique, in which excessive scan times are common in patients with irregular breathing.

The implemented iNAV postprocessing, including reconstruction, registration, and k‐space correction, takes approximately 200 to 300 milliseconds, depending on spatial resolution and size of ROI. Although this is acceptable for real‐time motion compensation, for which there is typically 600 to 1,000 ms between acquisitions (depending on heart rate), it precludes the use of prospective correction and gating. Therefore, gating mechanisms relying on prospectively adapting the k‐space segment based on respiratory position, such as PAWS 6 or respiratory ordered phase encoding 19, are incompatible with the current implementation of iNAV. Improved hardware and software design may reduce the latency of the iNAV processing. Nevertheless, it is unlikely that iNAV acquisition, utilizing the startup echoes that are in very close temporal proximity to the image readout, can be sufficiently short to enable prospective gating or tracking.

A further technical consideration that guided the design of the CRUISE gating strategy was the need to know, before the scan, how many navigator images to acquire for memory allocation purposes on the reconstruction computer. This in turn resulted in the choice of predefining a fixed respiratory gating efficiency of 50%, regardless of breathing pattern. This technical consideration precluded the use of a predefined gating window, and a direct comparison between the proposed gating strategy and conventional accept/reject gating using iNAV was not possible. Further work will focus on investigating additional stopping criterion for the gating algorithm, such as the iNAV variance or ensuring that the central k‐space lines are motion‐free. This may allow increased gating efficiency without compromising image quality.

### Limitations

This study has a number of limitations. The proposed algorithm is limited to rejection of end‐inspiratory positions. Outliers in end‐expiration may lead to a large motion range, although this never was observed in this study. Furthermore, it has been shown in a recent study that the use of an end‐expiratory reference navigator provides better image quality 20. However, in this implementation, the first iNAV acquisition was defined as the reference, which may lead to suboptimal image quality if it was acquired during inspiration. Further developments to the iNAV‐CRUISE technique will investigate the use of a navigator preparation phase to automatically define the iNAV‐CRUISE reference in end‐expiration. A further limitation is the comparison with alternative gating strategies, although technical challenges may preclude the use of prospective gating techniques with iNAV using the start‐up echoes.

## CONCLUSION

The proposed CMRA iNAV‐CRUISE approach out‐performs alternative navigator techniques such as selfNAV and the conventional respiratory motion compensation technique in healthy subjects. Although scan time was comparable, the image quality was improved using iNAV‐CRUISE. The technique can be readily deployed in a clinical setting. However, further patient studies are required to assess the diagnostic performance of the iNAV‐CRUISE technique.

## Supporting information

Additional supporting information may be found in the online version of this article

**Fig. S1**. Reformatted CMRA datasets acquired using iNAV‐CRUISE, selfNAV‐CRUISE, and dNAV‐2NSA.
**Fig. S2**. Coronary artery vessel sharpness for the right coronary artery (RCA), left anterior descending (LAD) artery and left circumflex artery (LCX) across five healthy subjects acquired using iNAV‐CRUISE, selfNAV‐CRUISE and dNAV‐2DNSA.
