# Supplementary material for: Coronary MR angiography using image‐based respiratory motion compensation with inline correction and fixed gating efficiency
Source: Magn Reson Med. 2017 Mar 20;79(1):416–22. doi: 10.1002/mrm.26678 (PMC5763408; doi:10.1002/mrm.26678)
Supplement: Supplementary file 1 — Fig. S1. Reformatted CMRA datasets acquired using iNAV‐CRUISE, selfNAV‐CRUISE, and dNAV‐2NSA. Fig. S2. Coronary artery vessel sharpness for the right coronary artery (RCA), left anterior descending (LAD) artery and left circumflex artery (LCX) across five healthy subjects acquired using iNAV‐CRUISE, selfNAV‐CRUISE and dNAV‐2DNSA. [file MRM-79-416-s001.docx]

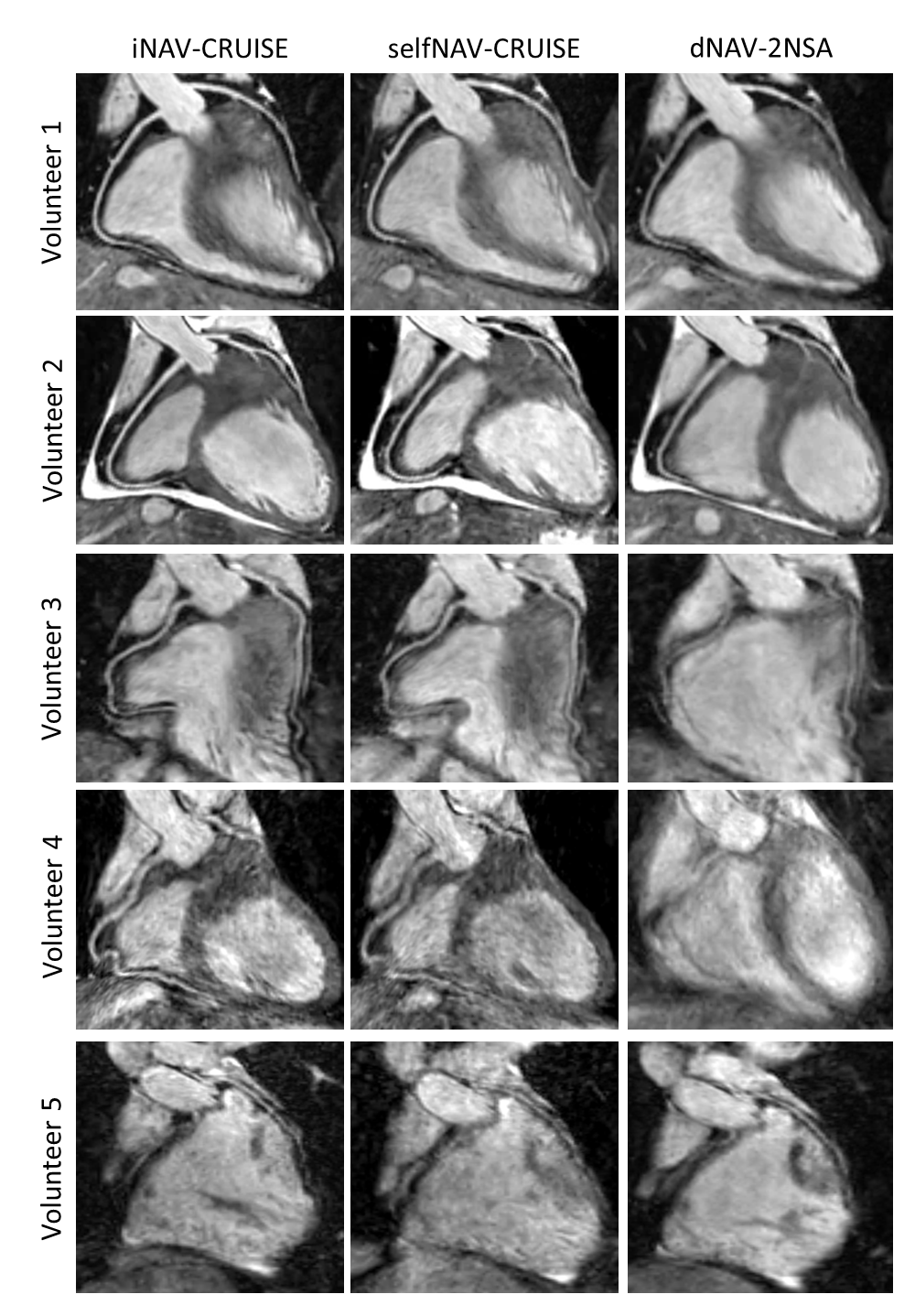


Supporting Figure S1. Reformatted CMRA datasets acquired using iNAV-CRUISE, selfNAV-CRUISE, and dNAV-2NSA.


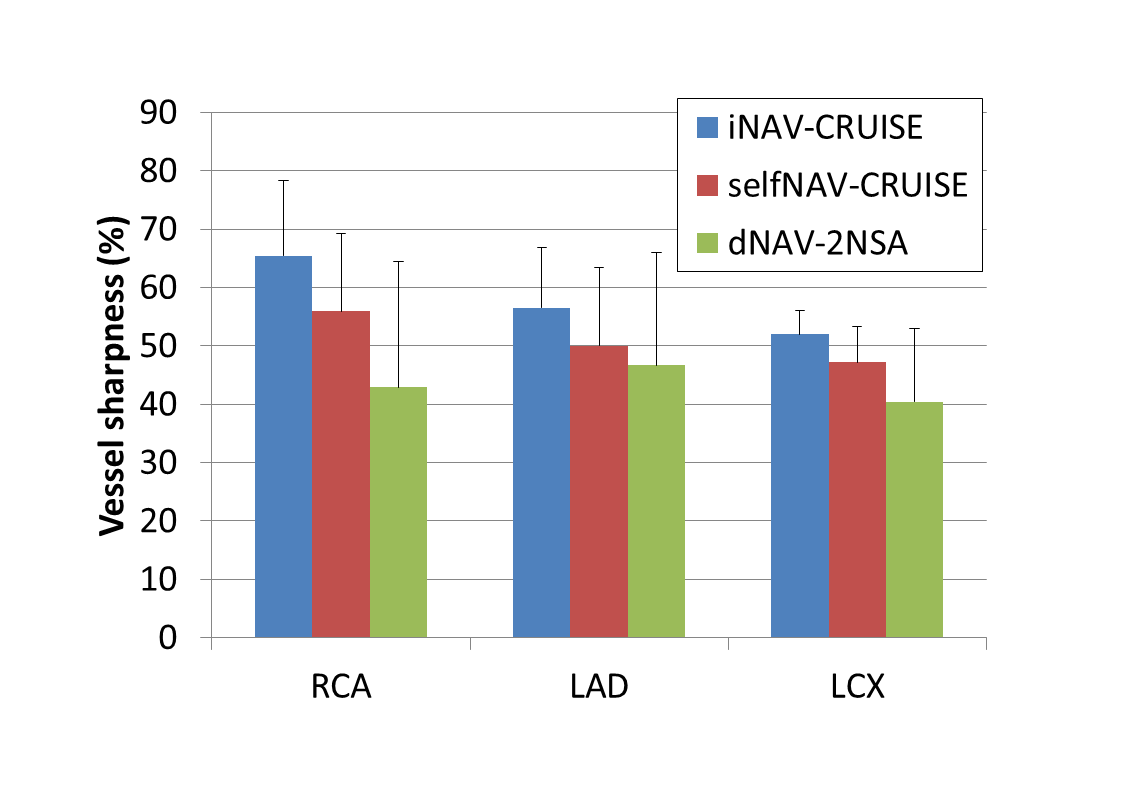


Supporting Figure S2. Coronary artery vessel sharpness for the right coronary artery (RCA), left anterior descending (LAD) artery and left circumflex artery (LCX) across five healthy subjects acquired using iNAV-CRUISE, selfNAV-CRUISE and dNAV-2DNSA.
